# Malaria Parasite Infection Compromises Control of Concurrent Systemic Non-typhoidal *Salmonella* Infection via IL-10-Mediated Alteration of Myeloid Cell Function

**DOI:** 10.1371/journal.ppat.1004049

**Published:** 2014-05-01

**Authors:** Kristen L. Lokken, Jason P. Mooney, Brian P. Butler, Mariana N. Xavier, Jennifer Y. Chau, Nicola Schaltenberg, Ramie H. Begum, Werner Müller, Shirley Luckhart, Renée M. Tsolis

**Affiliations:** 1 Department of Microbiology & Immunology, School of Medicine, University of California at Davis, Davis, California, United States of America; 2 Departamento de Clínica e Cirurgia Veterinária, Universidade Federal de Minas Gerais, Belo Horizonte, Minas Gerais, Brazil; 3 Department of Life Sciences & Bioinformatics, Assam University, Diphu Campus, Karbi Anglong, Assam, India; 4 Faculty of Life Sciences, University of Manchester, Manchester, United Kingdom; University of Toronto, Canada

## Abstract

Non-typhoidal *Salmonella* serotypes (NTS) cause a self-limited gastroenteritis in immunocompetent individuals, while children with severe *Plasmodium falciparum* malaria can develop a life-threatening disseminated infection. This co-infection is a major source of child mortality in sub-Saharan Africa. However, the mechanisms by which malaria contributes to increased risk of NTS bacteremia are incompletely understood. Here, we report that in a mouse co-infection model, malaria parasite infection blunts inflammatory responses to NTS, leading to decreased inflammatory pathology and increased systemic bacterial colonization. Blunting of NTS-induced inflammatory responses required induction of IL-10 by the parasites. In the absence of malaria parasite infection, administration of recombinant IL-10 together with induction of anemia had an additive effect on systemic bacterial colonization. Mice that were conditionally deficient for either myeloid cell IL-10 production or myeloid cell expression of IL-10 receptor were better able to control systemic *Salmonella* infection, suggesting that phagocytic cells are both producers and targets of malaria parasite-induced IL-10. Thus, IL-10 produced during the immune response to malaria increases susceptibility to disseminated NTS infection by suppressing the ability of myeloid cells, most likely macrophages, to control bacterial infection.

## Author Summary

Non-typhoidal *Salmonella* serotypes (NTS) most frequently cause diarrheal disease, which is self-limiting. However, in sub-Saharan Africa, NTS is one of the most common causes of life-threatening bloodstream infections. Individuals with these bloodstream infections frequently have an underlying condition such as HIV in adults or malaria in children. We used a mouse model to investigate why malaria predisposes children to invasive NTS infections. Our results implicate an anti-inflammatory response induced by malaria parasites via the cytokine IL-10 in promoting increased growth of bacteria that have disseminated from the intestine to other organs of the body. This response is beneficial in that it prevents death from malaria, but has an adverse effect on phagocytic cells, blocking their ability to control growth of bacteria that have disseminated from the intestine to other organs of the body.

## Introduction

In immunocompetent individuals, NTS serotypes are associated with gastroenteritis, a localized infection with low mortality that manifests as diarrhea, vomiting and intestinal cramping. However, immunocompromised individuals can develop life-threatening NTS bacteremia [1], [2]. Epidemiological associations suggest that the most common immunocompromising conditions predisposing to pediatric NTS bacteremia in sub-Saharan Africa are malnutrition and acute or recent malaria [1], [3]–[5]. The magnitude of the public health problem posed by NTS bacteremia is little publicized but it contributes considerably to morbidity and mortality in Sub-Saharan Africa [6]. For example, NTS are currently the most common blood isolates from children [3], [5], [7] and the second most common cause of pediatric meningitis in Malawi [8], resulting in mortality rates of approximately 50%, despite antibiotic therapy [9]. A factor complicating treatment of invasive NTS is the high prevalence of multidrug resistance [1], [10]–[13]. While the occurrence of NTS bacteremia in pediatric malaria patients is well documented, little is known about immunologic mechanisms underlying increased susceptibility to NTS bacteremia. This study was undertaken to identify mechanisms affecting the outcome of NTS infection in the setting of underlying malaria.

## Results

### Underlying *P. yoelii* infection leads to increased levels of systemic infection with *S.* Typhimurium, but reduced pyogenic inflammation

**Figure 1:**
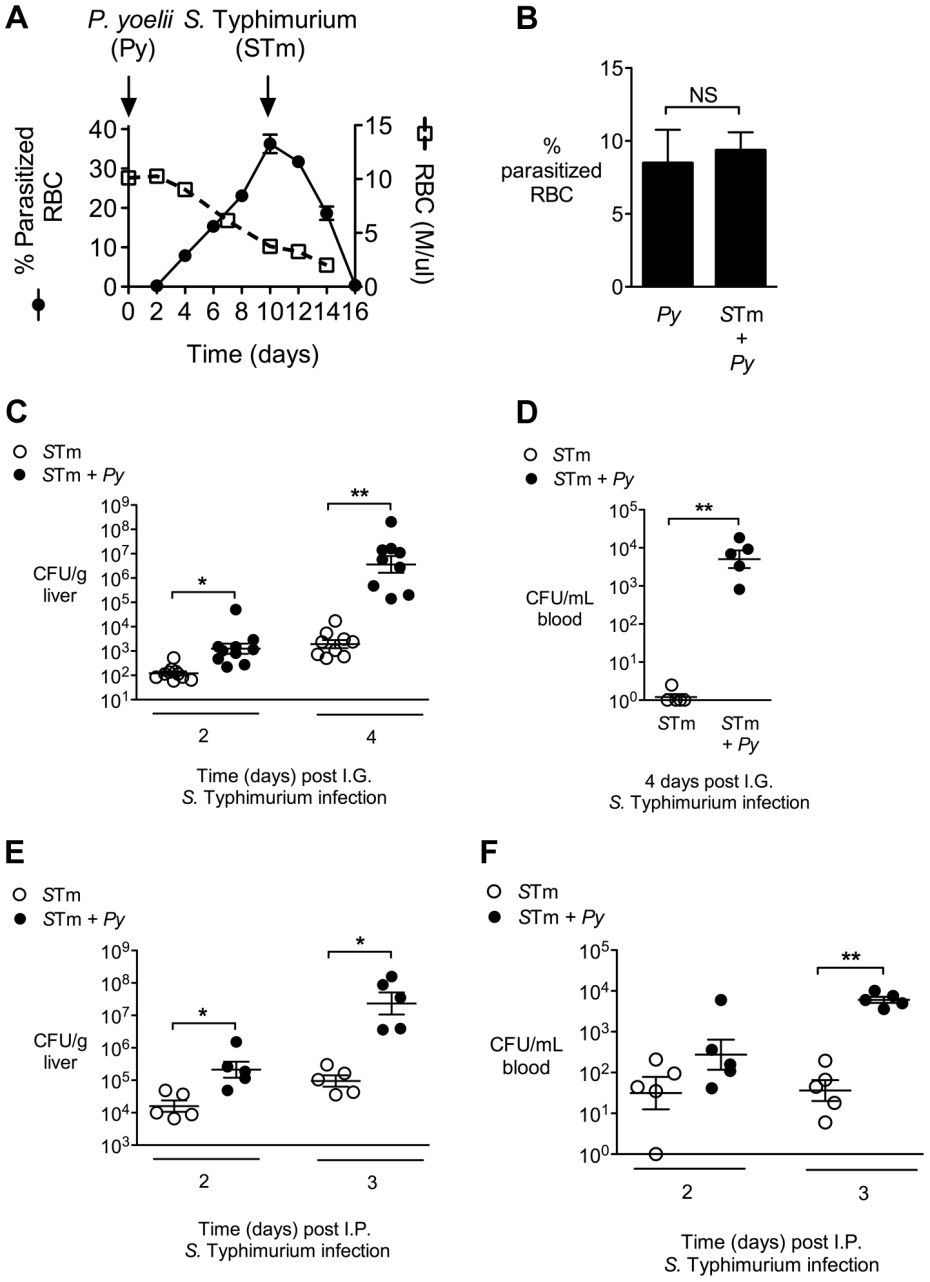
Increased systemic replication of *S.* Typhimurium (*S*Tm) during concurrent *P. yoelii* (*Py*) infection. **A,** Parasitemia and anemia in *P. yoelii*-infected mice (n = 4). Arrows indicate time points at which *P. yoelii* and *S.* Typhimurium were inoculated. **B,** Comparison of parasitemia at 14 d after *P. yoelii* infection in *P. yoelii*-infected mice and mice co-infected for 4 d with *S.* Typhimurium (n = 5). Data are shown as mean ± SEM. **C,** Colonization (CFU) of the liver at 2 and 4 days after IG infection of *P. yoelii*-infected or uninfected CBA mice with *S.* Typhimurium (n = 5–10). Results are from 2 independent experiments. **D,** CFU of *S.* Typhimurium in the blood 4 days after IG infection of CBA mice (n = 5). Panels, B, C and D are compiled from the two independent experiments. **E–F,** CFU in liver (**E**) and blood (**F**) at 2 and 3 days after IP infection of *P. yoelii*-infected or uninfected CBA mice (n = 5). Dots represent individual mice and bars represent the mean ± SEM. Statistical significance was determined using an unpaired Student's *t* test on log-transformed values and is indicated as *, *P*<0.05; **, *P*<0.01.

Since early studies on malaria patients demonstrated reduced responses to lipopolysaccharide (LPS) [14], and sensing of *S.* Typhimurium LPS via Toll-like receptor (TLR) 4 is crucial to control of NTS infection [15], we reasoned that malaria parasite infection might blunt innate immune responses required to control invasive bacteria. To test the idea that defective inflammatory responses in malaria could increase susceptibility to systemic infection, we used a mouse model to study the effects of malaria on inflammatory responses to NTS in a mouse strain (CBA) that was genetically resistant to lethal infection with both pathogens. To induce malaria in this model, mice were inoculated intraperitoneally (IP) with blood-stage *Plasmodium yoelii* subsp. *nigeriensis* (*P. yoelii*), a model for acute malaria. Maximal parasitemia and anemia were allowed to develop before intragastric (IG) inoculation with the NTS strain *Salmonella enterica* serotype Typhimurium 14028 (*S.* Typhimurium, **Fig. 1A**). Co-infection did not affect the levels of malaria parasite infection (**Fig. 1B**). However, co-infected mice exhibited increased bacterial loads of *S.* Typhimurium in the liver by 2 days post infection, as well as a more rapid increase in bacterial colonization between days 2 and 4, as compared to mice infected with *S.* Typhimurium alone (**Fig. 1C**). By 4 days post inoculation of *S.* Typhimurium, high numbers of bacteria were found in the blood of co-infected mice, while very few bacteria were detected in the circulation of mice infected only with *S.* Typhimurium (**Fig. 1C**). Co-infection with *P. yoelii* also resulted in elevated systemic colonization with a Malawian *S.* Typhimurium isolate, which belongs to a distinct sequence type circulating in sub-Saharan Africa [11] (**Fig. S1 in Text S1**). The rapid increase in systemic colonization of co-infected mice inoculated with *S.* Typhimurium by the IG route could result from increased intestinal permeability [16], as well as from increased replication of bacteria once they reach systemic sites. To distinguish between these possibilities, we performed an experiment in which mice were inoculated via the IP route with *S.* Typhimurium, to bypass the intestine (**Fig. 1E and 1F**). Co-infected mice inoculated IP with *S.* Typhimurium exhibited a similar rapid increase in bacteria within the liver (**Fig. 1E**) and blood (**Fig. 1F**) to mice inoculated via the IG route, suggesting that co-infection with malaria parasites promoted increased replication of *S.* Typhimurium at systemic sites.

**Figure 2:**
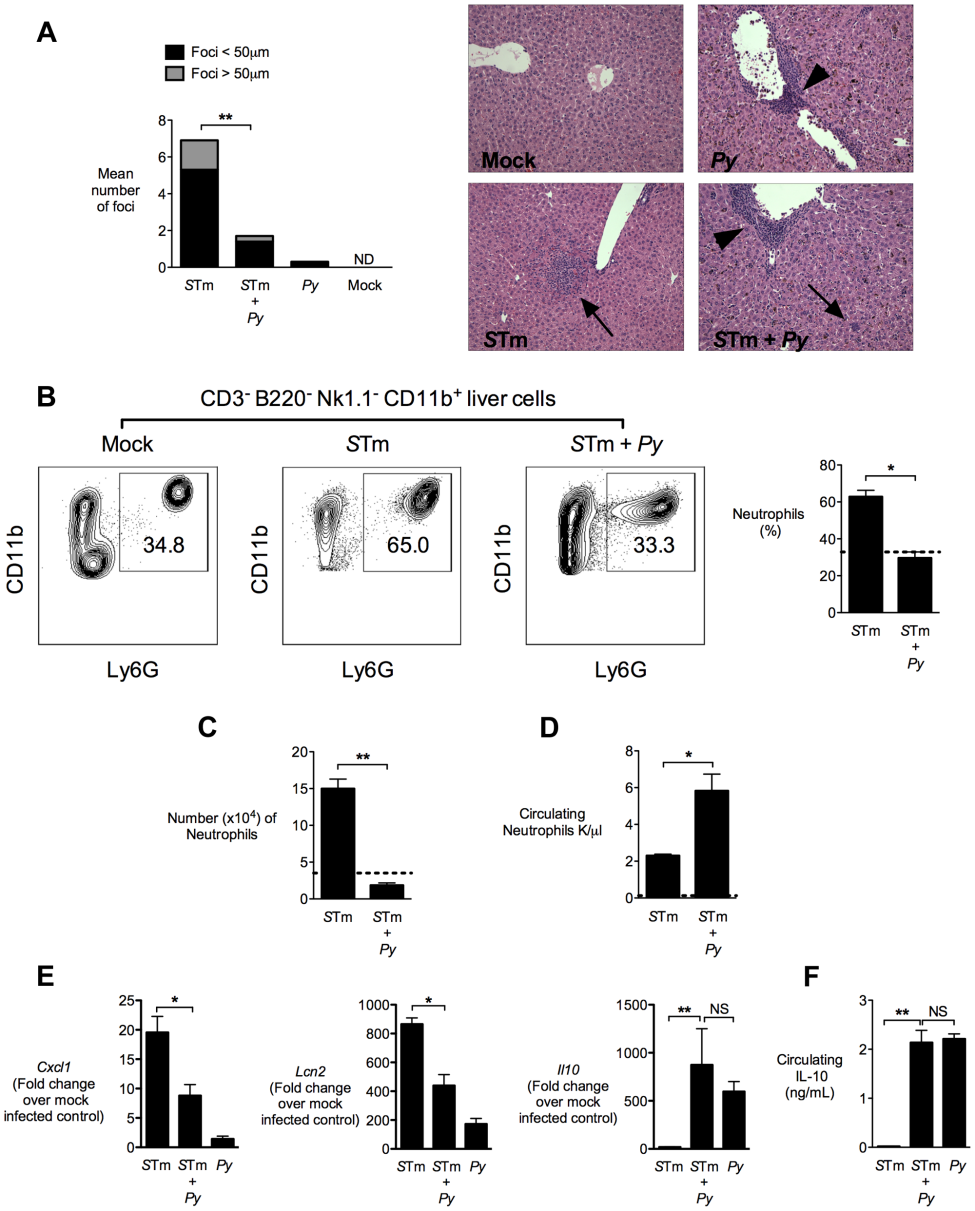
Decreased neutrophil-mediated pathology in the liver of co-infected mice. **A,** Quantification of inflammatory lesions in the liver and representative micrographs of H&E stained liver tissue from mock-infected mice, mice infected with *P. yoelii*, or co-infected mice 4 days after IG inoculation of *S.* Typhimurium in CBA mice (n = 5–10). Arrows represent pyogranulomatous lesions and arrowheads represent perivascular monocytic infiltrates. Results are from the experiment shown in figure 1B. **B,** Representative flow cytometry plots for neutrophil frequency in singlet live CD3^−^ B220^−^ NK1.1^−^ CD11b^+^ liver cells. Right panel shows quantification of neutrophils from *S.* Typhimurium and co-infected mice (n = 4–5). Dotted line represents mock-infected mice (n = 2). The gating strategy used for generation of these results is shown in **Fig. S2 in Text S1**. **C,** Number of neutrophils (singlet live CD3^−^ B220^−^ NK1.1^−^ CD11b^+^ Ly6G^+^) per 4×10^6^ liver cells determined by AccuCount beads. Results are from data shown in figure 2D. **D,** Number of circulating neutrophils (K/µl) determined by complete blood counts. Results are from data shown in figure 2C. **E,** Expression of *Cxcl1*, *Lcn2* and *Il10* in liver tissue of CBA mice 4 days after IG inoculation with *S.* Typhimurium (n = 4–9). Data expressed as fold change over mock-infected control. Results are from the experiment shown in figure 1B. **F,** Circulating IL-10 measured 2 days after IG infection with *S.* Typhimurium. Results are from the experiment shown in figure 1B. Data bars represent the mean +SEM. Statistical significance was determined using an unpaired Student's *t* test (**A–D, F**) or ANOVA with Tukey's post test (**E**) on log-transformed values and is indicated as *, *P*<0.05; **, *P*<0.01.

To determine whether underlying malaria affected inflammatory responses to disseminated NTS, histopathological comparison of inflammatory responses in the livers of *S.* Typhimurium-infected mice and mice co-infected with *P. yoelii* was performed. Despite nearly 10,000-fold higher bacterial numbers in the livers of the co-infected mice, this group had both significantly fewer microabscesses, and smaller-sized lesions, than mice infected only with *S.* Typhimurium. In a previous study, in which we inoculated mice simultaneously with *S.* Typhimurium and *P. yoelii* and evaluated inflammatory changes at d5 post infection, we found no effect of malaria parasite infection on *S.* Typhimurium-induced microabscess formation [17]. This difference between the previous study and our current results suggested that the blunting effect of malaria parasite infection on microabscess formation occurs later in the course of parasite infection. The blunting of microabscess formation observed in our current study suggested that a reduced inflammatory response to invasive *S.* Typhimurium in the liver could contribute to the increased bacterial load in this tissue during co-infection (**Fig. 2A**).

To determine whether the reduced size of inflammatory lesions was the result of decreased influx of neutrophils into the liver, we analyzed neutrophil infiltration by flow cytometry of cell suspensions from livers that were perfused with saline to remove circulating neutrophils (**Fig. 2B and 2C**). As expected, in livers of mice infected only with *S.* Typhimurium, a marked neutrophil influx was measured, compared to mock-infected controls. In contrast, in *P. yoelii*-infected mice, significantly fewer neutrophils were found in the liver after *S.* Typhimurium infection (**Fig. 2B and 2C**), which was consistent with the reduced inflammation observed by histopathology (**Fig. 2A**). The reduced infiltration of neutrophils into the liver of co-infected mice coincided with an increase in circulating neutrophils, compared to the mice infected with *S.* Typhimurium only (**Fig. 2D**). These results suggested that malaria parasite infection impaired recruitment of neutrophils that are needed to control *S.* Typhimurium infection. To test this idea, we measured expression of KC (*Cxcl1*), a CXC chemokine that recruits neutrophils to sites of infection (**Fig. 2E**). *Cxcl1* expression was strongly reduced in the liver of *P. yoelii* co-infected mice, compared to mice infected with *S.* Typhimurium alone (**Fig. 2E**), suggesting that reduced chemokine expression may be a factor contributing to impaired neutrophil recruitment. Expression of a second inflammation-associated gene, *Lcn2* encoding the siderophore-scavenging peptide Lipocalin-2, was also reduced in co-infected mice. This reduction of *Lcn2* expression could reflect reduction in two different cellular sources of Lipocalin-2, since Lipocalin-2 is produced both by *S.* Typhimurium-infected macrophages [18] and by neutrophils during malaria parasite infection [19], [20]. While *Salmonella*-induced pathology was blunted by co-infection, other pathologic changes induced by *P. yoelii*, including perivascular inflammation, hepatomegaly and induction of Tumor necrosis factor alpha (*Tnfa*) remained intact, or were increased by co-infection, compared to infection with *S.* Typhimurium alone (**Fig. 2** and **Fig. S3 in Text S1**).

### IL-10 elicited by malaria parasite infection contributes to increased systemic colonization

In malaria, induction of the immunoregulatory cytokine interleukin-10 (IL-10) has been shown to prevent excessive and potentially fatal pathology in murine models of cerebral malaria [21]–[24], and this important role of IL-10 has been corroborated by observational data from malaria patients [25]–[28]. In mice infected with *P. yoelii* or mice co-infected with *P. yoelii* and *S.* Typhimurium, but not in mice infected with *S.* Typhimurium alone, high levels of circulating IL-10 were measured (**Fig. 2F**). Similarly, in the liver, induction of *Il10* expression was observed in both *P. yoelii*-infected and co-infected mice, but not in mice infected only with *S.* Typhimurium (**Fig. 2E**). The similar levels of IL-10 detected in *P. yoelii* infection and co-infection indicated that the malaria parasites, rather than *S.* Typhimurium, were eliciting IL-10 production in this model (**Fig. 2E and 2F**).

**Figure 3:**
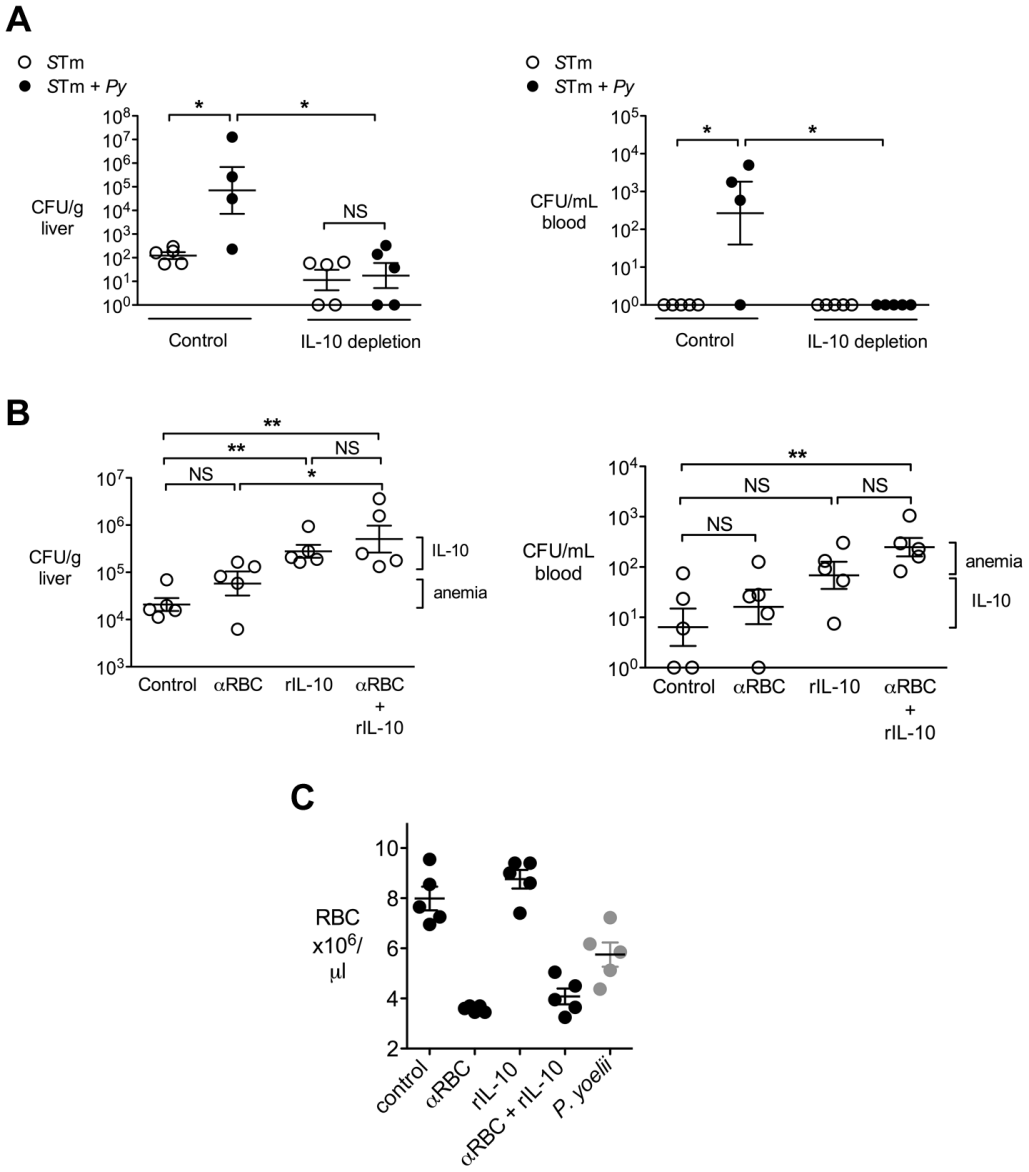
IL-10 contributes to increased systemic loads of *S.* Typhimurium in malaria parasite-infected CBA mice. **A,** *S.* Typhimurium and co-infected CBA mice as described in Fig. 1A, were depleted of IL-10 using a blocking antibody, or treated with a control antibody. At 2 d post-*S.* Typhimurium infection, CFU of *S.* Typhimurium were enumerated in liver and blood (n = 4–5). Statistical significance was determined using an unpaired Student's *t* test on log-transformed values, or a Mann-Whitney U test for groups that include all zero values, and is indicated as *, *P*<0.05; **, *P*<0.01. **B,** Mice were treated with either an anti-RBC antibody to induce anemia, recombinant IL-10 (rIL-10) or both. At 4 days post-*S.* Typhimurium infection, CFU were enumerated in liver and blood (n = 5). Dots represent individual mice and bars represent the mean ± SEM. Brackets indicate the contributions of anemia and IL-10 to increased bacterial colonization. **C,** Levels of anemia in experimental groups from (**B**) at 4 d post IG infection. The number of circulating RBC in mice infected with *P. yoelii* at 14 d after inoculation is given as a reference. Statistical significance was determined using one-way ANOVA with a Tukey's post test *, *P*<0.05; **, *P*<0.01.

To determine whether parasite-induced IL-10 contributed to the increased systemic loads of *S.* Typhimurium in co-infected mice, we blocked the effects of IL-10 using a neutralizing antibody (**Fig. 3A**). In co-infected mice, neutralization of IL-10 reduced bacterial loads in both the liver and in the blood, suggesting a contribution of this cytokine to the increased systemic levels of *S.* Typhimurium during co-infection. In contrast, in mice infected with *S.* Typhimurium only, IL-10 depletion had no effect on bacterial colonization, suggesting that the effect of IL-10 blocking in this experiment was specific to co-infection (**Fig. 3A**).

### IL-10 and anemia act together to increase susceptibility to systemic *S.* Typhimurium infection

In the experiments shown above, mice infected with *P. yoelii* developed severe anemia (**Fig. 1A**) as well as high IL-10 levels (**Fig. 2F**). Studies in Africa that established a link between NTS bacteremia and malaria reported an association between pediatric NTS bacteremia and severe malarial anemia or recent malaria [3], [29], [30]. To determine whether parasite-induced anemia could have an additive effect with parasite-induced IL-10, we determined the effects of anemia and IL-10, both individually and in combination, on susceptibility to systemic *S.* Typhimurium infection (**Fig. 3B**). Mice were administered recombinant IL-10 (rIL-10), or anti-red blood cell (α-RBC) IgG to induce anemia, or received both treatments. All mice treated with α-RBC antibodies developed severe anemia comparable to that induced by *P. yoelii* infection (**Fig. 3C**). However, when compared to mock-treated controls, a significant increase in bacteremia was observed only in mice treated with both rIL-10 and α-RBC antibodies (**Fig. 3B** **, right panel**). Similarly, compared to treatment with α-RBC antibodies, an additive effect of IL-10 treatment was observed on colonization of the liver (**Fig. 3B** **, left panel**). This interpretation of an additive effect of both factors would be consistent with our previous results suggesting that induction of anemia resulted in lower levels of NTS bacteremia than parasite infection [17].

### IL-10 elicited by malaria parasite infection blunts hepatic inflammatory responses induced by *Salmonella*

To determine the effect of IL-10 on inflammation induced by *S.* Typhimurium, we used mice deficient for *Il10*, which were on a C57BL/6 strain background. An important difference between the C57BL/6 strain and the CBA strain used for our previous experiments is that C57BL/6 mice have a defective *Slc11a1* (also known as *Nramp1*) allele, which reduces the ability of resident<1?tlb 10.5pt?> macrophages to control systemic *S.* Typhimurium infection [31]–[35]. Experiments with *P. chabaudi* showed that CBA and C57BL/6 mice are both able to control malaria parasite infection, therefore use of the C57BL/6 strain was not expected to alter susceptibility to NTS bacteremia via an effect on malaria parasite infection [36].

**Figure 4:**
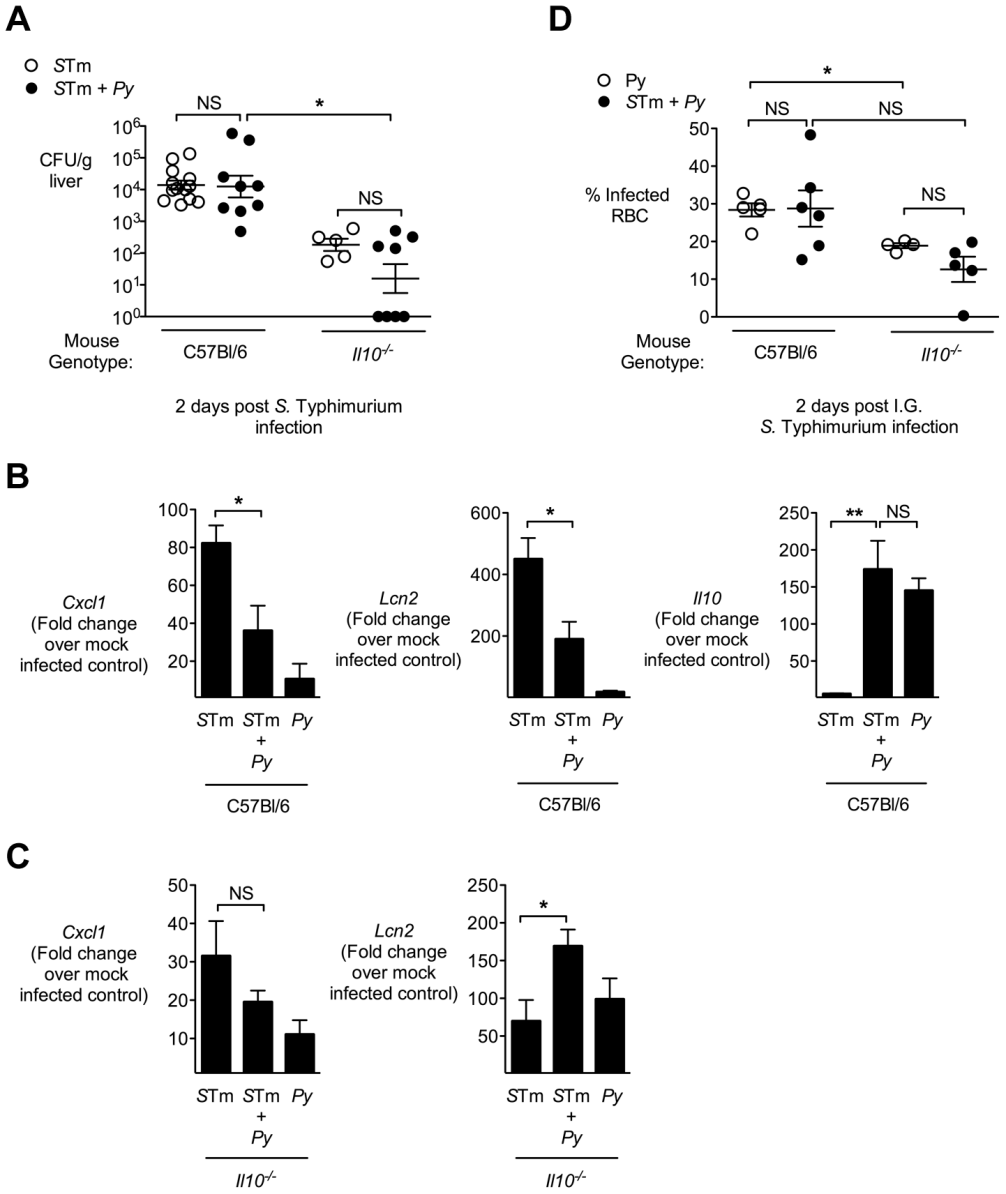
IL-10 contributes to blunting of neutrophil chemokines in livers of malaria parasite-infected C57BL/6 mice. **A,** Mice deficient for IL-10 (*Il10^−/−^*) or controls (C56BL/6) were infected with either *S.* Typhimurium or co-infected as described in Fig. 1A. At 2 days post-*S.* Typhimurium infection, CFU of *S.* Typhimurium was enumerated in liver (n = 5–13). Dots represent individual mice. **B,** Expression of *Cxcl1*, *Lcn2* and *Il10* in liver tissue of control mice, 2 days after *S.* Typhimurium infection (n = 5–13). Results are from data shown in figure 4A. **C,** Expression of *Cxcl1* and *Lcn2* in liver tissue of *Il10^−/−^* mice, 2 days after *S.* Typhimurium infection (n = 5–8). Results are from data shown in figure 4A. **D,** Effect of co-infection with *S.* Typhimurium in C57BL/6 and *Il10^−/−^* mice on malaria parasitemia. C57BL/6 and *Il10^−/−^* and mice were inoculated IP with blood stage *P. yoelii*, then at day 10, were inoculated IG with *S.* Typhimurium. Parasitemia was determined at day 12 (2 days after *S.* Typhimurium infection). Bars represent the mean +SEM (n = 4–6). Data from control mice are compiled from 3 independent experiments and *Il10^−/−^* from 1 experiment. Statistical significance was determined using an unpaired Student's *t* test (**A**, **D**) or one-way ANOVA with Tukey's post test (**B**, **C**) on log-transformed values and is indicated as *, *P*<0.05; **, *P*<0.01.

Co-infection of C57BL/6 mice, with the same dose of bacteria and parasites used in CBA mice, resulted in 10–100-fold higher levels of *S.* Typhimurium in the liver at 2d after inoculation with *S.* Typhimurium, compared to CBA mice (**Fig. 4A** and **Fig. 1B**). In this set of experiments, mice progressed rapidly to lethal morbidity between days 2–3. Further, at 2d post infection, in contrast with the CBA mice, no difference in colonization with *S.* Typhimurium was observed in the liver of C57BL/6 mice between groups infected only with *S.* Typhimurium and co-infected with *P. yoelii* and *S.* Typhimurium (**Fig. 4A**). Compared to control mice co-infected with *P. yoelii*, the *Il10^−/−^* mice had reduced loads of *S.* Typhimurium in the liver (**Fig. 4A**). Since the levels of bacterial colonization between *S.* Typhimurium-infected mice and malaria parasite co-infected mice were similar, we examined the effect of malaria parasite co-infection on inflammatory responses to *S.* Typhimurium in the liver. In a similar manner to what was observed in CBA mice (**Fig. 2**), the co-infected C57BL/6 mice exhibited blunting of *Cxcl1* and *Lcn2* expression (**Fig. 4B**). However in co-infected *Il10^−/−^* mice this blunting of proinflammatory cytokine expression was absent (**Fig. 4C**). As reported previously for *P. chabaudi* infection [37], the co-infected *Il10^−/−^* mice had lower *P. yoelii* parasitemia (**Fig. 4D**), suggesting that part of the effect of IL-10 deficiency on inflammatory responses may be an indirect effect of reduced parasite infection. These results suggested that induction of *Il10* expression by malaria parasite infection led directly or indirectly to blunting of inflammatory responses elicited by *S.* Typhimurium in the liver.

### Malaria parasite-induced IL-10 acts on myeloid cells to increase systemic NTS infection

**Figure 5:**
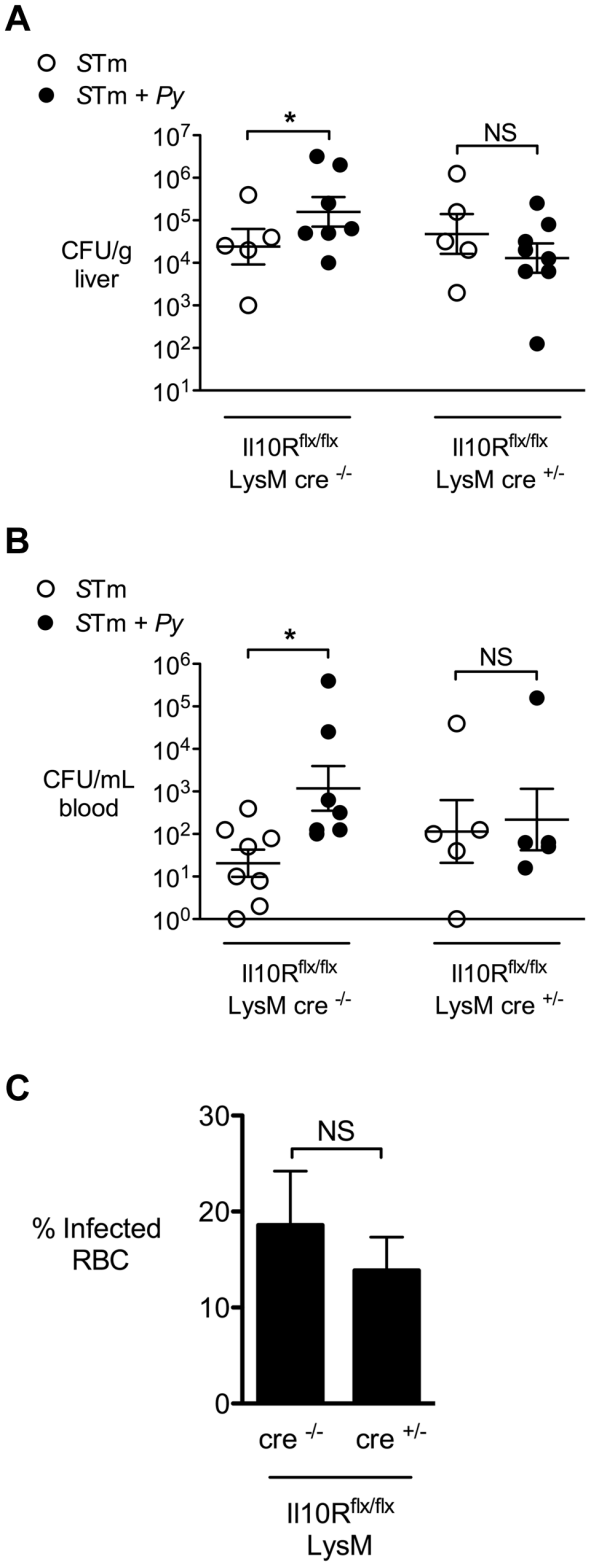
Malaria parasite-induced IL-10 acts on myeloid cells. Mice conditionally deficient for IL-10 receptor (Il10R) on myeloid cells (*Il10R^f/f^ LysM-cre*) or Cre-negative littermate controls were infected with *S.* Typhimurium or co-infected with *S.* Typhimurium and *P. yoelii*. Bacterial colonization was assessed 2 days after IP inoculation with *S.* Typhimurium and 12 days after *P. yoelii* inoculation (n = 5–8). **A,** Colonization of liver tissue by *S.* Typhimurium. **B,** *S.* Typhimurium bacteremia. **C,** Parasitemia in Cre^+/−^ and Cre^−/−^ mice co-infected with *P. yoelii* and *S.* Typhimurium. Dots represent individual mice and bars represent the mean ± SEM. Data are compiled from 2 independent experiments. Statistical significance was determined using an unpaired Student's *t* test on log-transformed values and is indicated as *, *P*<0.05; **, *P*<0.01.

*S.* Typhimurium is found within liver macrophages (also known as Kupffer cells) during infection [38], and these cells play an important role in control of systemic *S.* Typhimurium burden [15], [39]. We therefore hypothesized that the IL-10-dependent increase in systemic colonization of *S.* Typhimurium during co-infection could result from an effect of IL-10 on resident macrophages [40]. To determine whether macrophage responsiveness to IL-10 promotes disseminated NTS infection, we bred mice that were deficient for IL-10 receptor (IL-10R) expression on monocytes/macrophages and neutrophils (LysM-cre^+/−^×IL10R1^flox/flox^) on a C57BL/6 strain background. Groups of LysM-cre^−/−^ littermate controls and LysM-cre^+/−^ (conditionally IL-10R-deficient) mice were then inoculated with *S.* Typhimurium by the IP route or co-infected with *P. yoelii* and *S.* Typhimurium. Since our earlier results showed that the co-infected C57BL/6 mice experienced rapid lethal morbidity and high systemic loads of *S.* Typhimurium (**Fig. 4**), we reduced the inoculum of both *S.* Typhimurium and *P. yoelii* in these experiments, as described in the Materials and Methods. At 2 days post *S.* Typhimurium infection, the LysM-cre^−/−^ controls had significantly more bacteria in both the liver (**Fig. 5A**) and in the blood (**Fig. 5B**). In contrast, co-infection with *P. yoelii* had no effect on the ability of the mice deficient for IL-10R expression in myeloid cells to control systemic *S.* Typhimurium infection (**Fig. 5A and 5B**). The improved control of systemic *S.* Typhimurium infection in the conditionally IL-10R-deficient mice did not appear to be an indirect effect of reduced parasitemia, since at day 14 post *P. yoelii* infection, no significant difference in parasitemia was observed between the LysM-cre^+/−^ and the LysM-cre^−/−^ control groups (**Fig. 5C**).

### Concurrent malaria parasite infection alters gene expression in tissue macrophages

**Figure 6:**
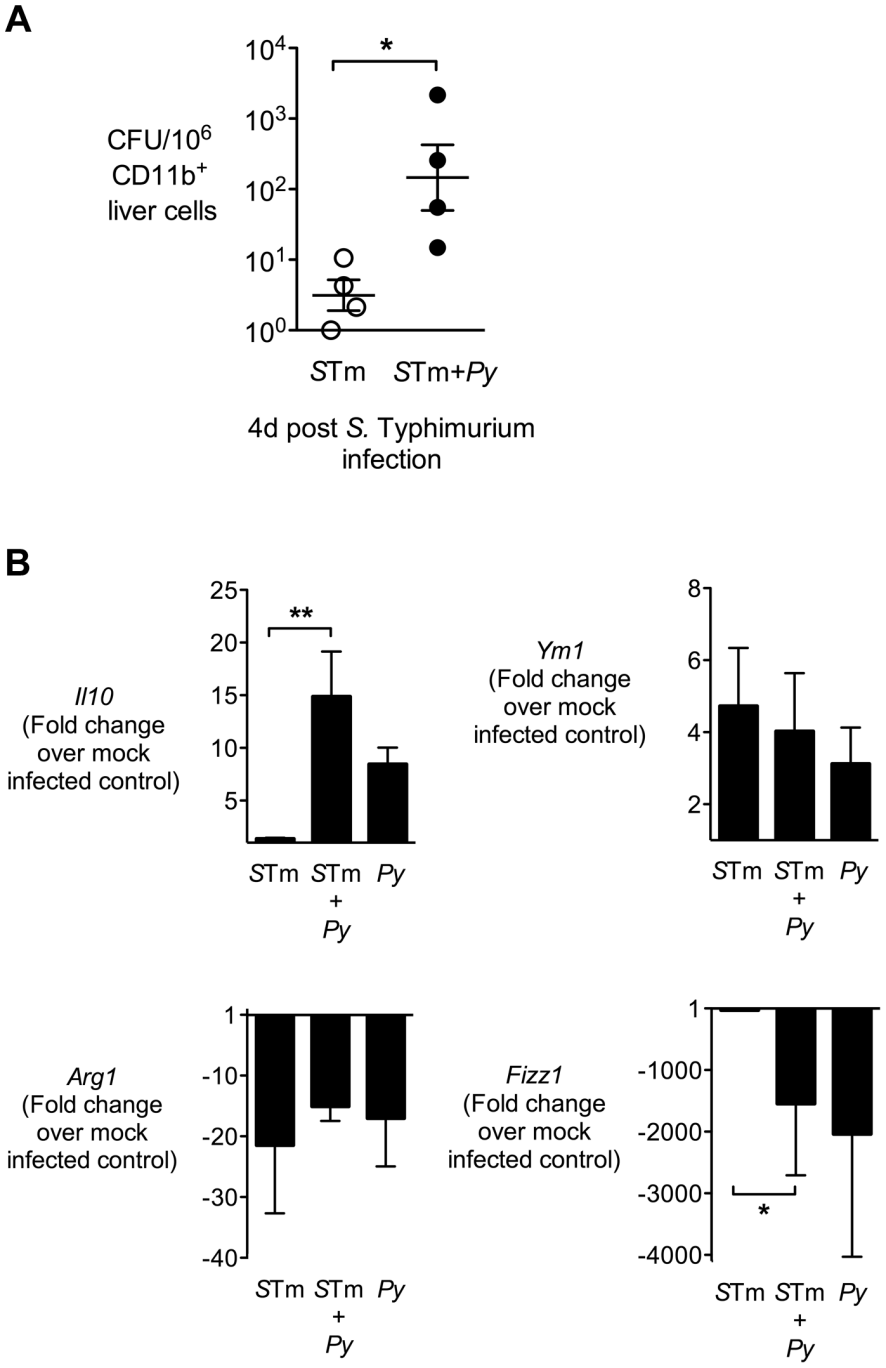
Liver CD11b^+^ macrophages exhibit increased association with *S.* Typhimurium and an altered phenotype during malaria parasite co-infection. **A,** CD11b^+^ liver cells were enriched by positive selection from *S.* Typhimurium-infected and co-infected CBA mice at 4 days post-*S.* Typhimurium infection (n = 4). CFU of *S.* Typhimurium was enumerated from 10^6^ CD11b^+^ liver cells. CD11b^+^ liver cells, **B,** Expression of *Il10*, *Ym1*, *Fizz1*, and *Arg1* in CD11b^+^ liver cells was determined by qRT-PCR. Data are expressed as fold change over mock-infected control. All mice were co-infected as described in Fig. 1A. Dots represent individual mice and bars represent the mean ± SEM. Statistical significance was determined using an unpaired Student's *t* test (**A**) or one-way ANOVA with Tukey's post test (**B**) on log-transformed values and is indicated as *, *P*<0.05; **, *P*<0.01.

During chronic infection *S.* Typhimurium has been found to persist preferentially within M2, or alternatively activated macrophages [41], as well as in hemophagocytic macrophages expressing M2 activation markers [42], therefore we tested the idea that malaria might polarize liver macrophages to an M2 phenotype. To this end, we analyzed expression profiles of alternative activation markers in the CD11b^+^ cell-enriched liver fraction of CBA mice infected IG with *S.* Typhimurium only, or co-infected with *S.* Typhimurium at 14 d after *P. yoelii* infection. At 4 d after IG *S.* Typhimurium infection, macrophages were positively selected from liver homogenates using CD11b-immmunomagnetic beads (**Fig. 6**). The CD11b^+^ fraction from co-infected mice contained 50-fold more bacteria than in mice infected with *S.* Typhimurium only, suggesting that at least part of the increased bacterial load in the liver is associated with macrophages (**Fig. 6A**).

Analysis of gene expression in the CD11b^+^ cell fraction revealed these cells did not have elevated expression of typical alternatively activated macrophage markers such as *Fizz1*, *Arg1*, or *Ym1* (**Fig. 6B**) as well as mannose receptor (not shown). In contrast, *Il10*, a marker of regulatory macrophages, was expressed more highly in the CD11b^+^ liver cell fraction of mice infected with *P. yoelii* or co-infected with *P. yoelii* and *S.* Typhimurium (**Fig. 6B**), suggesting that malaria may polarize liver macrophages to a M2-like regulatory phenotype [43].

### Macrophages are a source of IL-10 during co-infection

**Figure 7:**
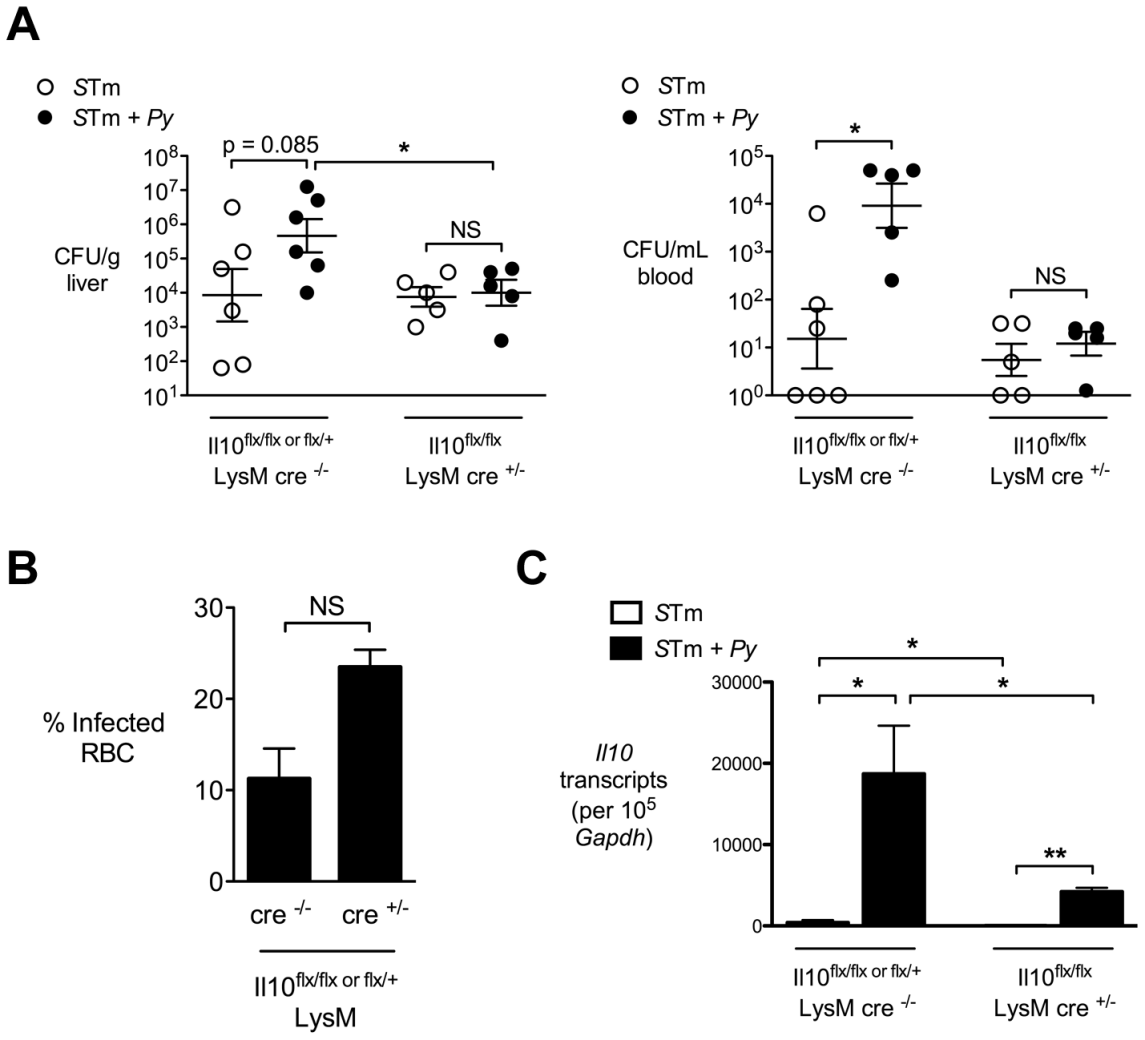
Macrophage/neutrophil derived IL-10 contributes to increased systemic *S.* Typhimurium burden during concurrent malaria parasite infection. Mice conditionally deficient for IL-10 expression on myeloid cells (*Il10^f/f^ LysM-cre*) or Cre-negative littermate controls were infected with *S.* Typhimurium or co-infected with *S.* Typhimurium and *P. yoelii*. Bacterial colonization was assessed 2 days after IP inoculation with *S.* Typhimurium and 12 days after *P. yoelii* inoculation (n = 5–6). **A,** Colonization of liver tissue (left panel) and blood (right panel) by *S.* Typhimurium. **B,** Parasitemia in Cre^+/−^ and Cre^−/−^ mice co-infected with *P. yoelii* and *S.* Typhimurium **C,**. Expression of *Il10* in livers of *Il10^f/f^ LysM-cre* mice or littermate controls at 12 after *P. yoelii* infection and 2 days after *S.* Typhimurium infection. Bars represent the mean +SEM (n = 5). Dots represent individual mice and bars represent the mean ± SEM. Data are compiled from 2 independent experiments. Statistical significance was determined using an unpaired Student's *t* test on log-transformed values and is indicated as *, *P*<0.05; **, *P*<0.01.

To determine the importance of macrophage IL-10 production for malaria-mediated susceptibility to disseminated infection, we bred mice that were conditionally deficient for IL-10 production by cells of the myeloid lineage, which includes neutrophils and monocytes/macrophages. To this end, C57BL/6 mice expressing a LysM-cre allele (specific for myeloid cells) were bred to mice homozygous for a floxed *Il10* allele (IL10^flox/flox^) [44]. Conditionally IL-10 deficient mice, or their respective Cre-negative littermate controls were co-infected with *S.* Typhimurium and *P. yoelii* as described above (**Fig. 7**). At 2 days post IP infection with *S.* Typhimurium, the co-infected, Cre-negative littermate controls had on average 50-fold higher (*P* = 0.08) loads of *S.* Typhimurium in the liver compared to mice infected with *S.* Typhimurium only, and a 1000-fold higher burden of *S.* Typhimurium in the circulation (*P*<0.05; **Fig. 7A**). In contrast, in the *cre*-positive mice that were conditionally deficient for IL-10 production by macrophages and neutrophils, no difference in *S.* Typhimurium colonization was observed between co-infected and *S.* Typhimurium-infected groups of mice. Unlike what we observed with completely IL-10 deficient mice (**Fig. 4D**), the improved ability of the conditionally IL-10 deficient mice to control systemic *S.* Typhimurium infection could not be attributed to an indirect effect of a reduction in *P. yoelii* infection, since the conditionally deficient mice actually had a nonsignificant increase in circulating parasites (**Fig. 7B**). Elimination of macrophage/neutrophil IL-10 production led to a striking reduction of *Il10* expression in the liver of co-infected mice, indicating that in this tissue, *LysM*-expressing cells are the major producers of IL-10 (**Fig. 7C**). Together, these results suggested that malaria parasite-induced production of IL-10 by macrophages and/or neutrophils is a mechanism that compromises control of systemic bacterial infection in the co-infected mice.

Since the LysM-cre allele used to delete *Il10* in the conditional knockout mice is expressed in both monocytes/macrophages and neutrophils our results suggested that production of IL-10 by both cell types could compromise immunity to *S.* Typhimurium in the mouse. However, the lack of neutrophil influx into tissues of *S.* Typhimurium and *P. yoelii*-coinfected CBA mice (**Fig. 2B**) as well as a lack of *Il10* induction in bone marrow neutrophils during *P. yoelii* infection (**Fig. S4 in Text S1**) suggest that macrophage-derived IL-10 is more important than neutrophil-derived IL-10 in suppressing control of systemic *S.* Typhimurium infection in the malaria parasite-infected mice.

In the context of murine malaria models, CD4 T cells were shown to be an important cellular source of immunomodulatory IL-10 [37], [45]. We therefore asked whether IL-10 produced by CD4 T cells contributed to increased infection with *S.* Typhimurium. To this end, IL10^flox/flox^×CD4-cre mice (conditionally deficient for IL-10 production by T cells; [46]), and their respective littermate controls were co-infected with *S.* Typhimurium and *P. yoelii* as described above. Similar to a previous report [37], mice conditionally for IL-10 production by CD4 T cells had a trend for lower parasitemia during co-infection (**Fig. S5A in Text S1**). However in the liver, the CD4 T cell-specific defect in IL-10 had no effect on expression of *Il10* in tissue, suggesting that at this site, T cells are not a major source of IL-10 during co-infection. (**Fig. S5B in Text S1**). Consistent with this result, IL-10 production by CD4 T cells had no effect on control *S.* Typhimurium infection during malaria co-infection (**Fig. S5C in Text S1**).

In summary, our results show that IL-10 induced by malaria parasite infection acts on phagocytic cells to shift their activation state toward a regulatory, IL-10 producing phenotype. As a result, these cells lose their ability to control *S.* Typhimurium infection. This compromise of tissue macrophage function may contribute to the increased susceptibility of individuals with malaria to disseminated NTS infection.

## Discussion

The results of our study show that in the context of malaria parasite infection, the host's ability to control systemic *S.* Typhimurium infection is compromised. One factor contributing to increased infection levels of *S.* Typhimurium during co-infection with malaria is induction of IL-10. In murine malaria models, the protective role of IL-10 in dampening excessive inflammation is well-established [21]–[23]. The sources of immunomodulatory IL-10 in the *P. yoelii* and *P. chabaudi* malaria models are populations of CD4 T cells [17], [37], [45].

Thus, in murine malaria models, production of IL-10 serves to limit damage caused by the inflammatory response to malaria parasites. Induction of IL-10 expression by intestinal helminths has also been shown to have beneficial effects by inhibiting colitis in a murine model of inflammatory bowel disease [47]. While in the context of co-infection with malaria parasites and NTS, immunoregulation by IL-10 could prevent inflammatory pathology and resulting organ damage, a clear downside of this regulation is a reduced ability to limit bacterial burden at systemic sites (**Fig. 2**). An important target of parasite-induced IL-10 was cells of the myeloid lineage, most likely macrophages. A shift was observed in the phenotype of macrophages within the liver toward a population that produced IL-10, which would be consistent with a subset of M2 or alternatively activated macrophages [43], [48]. IL-10 production by macrophages was also an essential determinant of susceptibility to systemic infection (**Fig. 7** and **Fig. S6 in Text S1**), suggesting that in the context of co-infection, macrophages both produce and respond to IL-10. These results are consistent with a previous study that identified IL-10 producing macrophages in the spleen during *P. chabaudi* infection [37]. Previous studies have implicated heme, released via lysis of infected erythrocytes, or hemozoin, the product of hemoglobin degradation by malaria parasites, in induction of IL-10 expression by macrophages, but the mechanism by which this occurs is unknown [49], [50].

Since *S.* Typhimurium is an intracellular pathogen that resides within macrophages during infection, IL-10 produced by macrophages might act locally on tissue macrophages to generate a more permissive niche for intracellular *S.* Typhimurium infection. Both alternatively activated macrophages and hemophagocytic macrophages express an M2 phenotype that renders them more permissive for intracellular replication of *S.* Typhimurium [41], [42], [51], suggesting that in the setting of malaria, IL-10 induced by parasite infection could contribute to a shift in macrophage activation state, thereby providing a more favorable environment for *S.* Typhimurium infection. At the cellular level, multiple effects of the tissue cytokine environment could promote increased intracellular *S.* Typhimurium infection. For example, compared to M1 or classically activated macrophages, M2 macrophages undergo a metabolic shift that increases intracellular glucose availability, and this has been shown to promote intracellular growth of both *S.* Typhimurium and *Brucella abortus* [41], [52]. Further, *in vitro* studies have shown that blocking of IL-10 in cultured macrophages leads to an increased localization of two other intracellular pathogens, *Mycobacterium tuberculosis* and *Brucella abortus*, to LAMP1-positive compartments, and this corresponded with reduced intracellular replication of these bacteria [53], [54]. These studies, together with a study demonstrating increased phagolysosomal fusion of *S.* Typhimurium-containing vacuoles after IL-10 neutralization in trophoblast-like cell cultures [55], suggest that *in vivo*, IL-10 induced by malaria parasite infection might alter membrane traffic in tissue macrophages as well. It will be interesting to perform a more detailed characterization of functional changes to the macrophage population *in vivo* that are induced by malaria parasite infection.

This IL-10 dependent defect in macrophage function is likely to act together with additional immune defects caused by malaria. Recently it was shown by Cunnington et al., that hemolysis caused by malaria parasite infection suppresses neutrophil function [56], [57]. Neutrophils play an important role in controlling systemic *Salmonella* infection, especially in the liver [58]. *S.* Typhimurium replication within macrophages induces pyroptosis, a form of inflammatory cell death, resulting in its release from macrophages [59]–[61]. Uptake and killing of these extracellular bacteria by neutrophils is critical in preventing their spread to other cells [62]. Thus IL-10-induced suppression of neutrophil recruitment and macrophage function likely synergize with this neutrophil defect to increase susceptibility to disseminated NTS infection in the setting of malaria (**Fig. S6 in Text S1**). Additional parasite-induced changes to host physiology that have been documented in malaria, including depletion of complement and reduction in intestinal barrier function, may also play a role in promoting systemic NTS disease [16], [63], [64].

In conclusion, our results provide evidence that malaria parasite-induced IL-10, a response that limits pathology in the context of malaria, compromises innate immune responses to *S.* Typhimurium, a prevalent invasive bacterial infection, by compromising the function of phagocytic cells. Further studies will be needed to determine whether this mechanism occurs in human malaria patients and whether it compromises responses to other invasive pathogens.

## Materials and Methods

Additional methods are provided in Text S1.

### Ethics statement

Experiments with mice were carried out in strict accordance with the recommendations in the Guide for Care and Use of Laboratory Animals of the National Institute of Health and were approved by the Institutional Animal Care and Use Committees at the University of California at Davis under protocols 15702 and 16597.

### Mouse strains

Specific pathogen free 6–8 week-old female CBA/J, C57BL/6J and C57BL/6J IL10^−/−^ (B6.129P2-*Il10^tm1Cgn^*/J; [65]) mice were purchased from the Jackson Laboratory (Bar Harbor, Maine). Mice with a floxed *Il10* allele (B6-*Il10^tm3Cgn^*; [44]), *Il10R* allele (B6-*Il10ra^tm1.1Jack^*, [66]) and LysM-cre mice or CD4-cre mice, [67] used to generate mice conditionally deficient for synthesis of IL-10 or IL-10R1, were described previously. For all conditionally deficient mice, Cre-negative littermates carrying the floxed allele of interest were used as controls. Mice were maintained under specific pathogen-free conditions by the UC Davis Center for Laboratory Animal Science, receiving irradiated rodent chow and sterile drinking water ad libitum. IL10-deficient mice were evaluated before use for signs of inflammatory bowel disease, and mice with rectal prolapse, abnormal fecal pellets or failure to gain weight at the normal rate for C57BL/6 mice were excluded from the study. Whole blood was collected with heparinized syringes and complete blood counts were analyzed by the UC Davis Comparative Pathology Laboratory.

### *Plasmodium yoelii nigeriensis* (*P. yoelii*)

Parasite stocks were obtained from the Malaria Research and Reference Reagent Resource and the species was confirmed by DNA sequencing of merozoite surface protein-1 (MSP-1) [17]. Parasite stocks were made by passage in CD-1 mice. For co-infection experiments in CBA and C57BL/6, mice were inoculated intraperitoneally (IP) on day 0 with approximately 4×10^7^ infected red blood cells (iRBCs) in 0.1 ml of saline. For co-infection experiments in conditionally deficient strains, mice were inoculated with approximately 4×10^6^ iRBCs. Mock-infected controls were injected IP with an equivalent amount of blood from uninfected CD-1 mice.

### *Salmonella enterica* serotype Typhimurium

*S.* Typhimurium strain IR715 (pHP45Ω), resistant to nalidixic acid, ampicillin and streptomycin, was used for this study [68], [69]. To ensure consistent infection of resistant CBA mice with *S.* Typhimurium, intragastrically (IG) inoculated mice received 20 mg of streptomycin (Sigma) by gavage 24 h prior to infection. Mice received either 0.1 ml of sterile Luria-Bertani (LB) broth or 1×10^8^ colony forming units (CFU) of *Salmonella* in 0.1 ml of LB broth by gastric gavage. Inocula were cultured for 16 h aerobically with selective pressure (50 mg/L carbenicillin) at 37°C. For some experiments, *S.* Typhimurium strain D23580, a multidrug-resistant bloodstream isolate from a Malawian child with malaria and NTS bacteremia [11], was used. For IP inoculation, mice received 100–500 CFU of *S.* Typhimurium in phosphate-buffered saline (PBS) or an equivalent volume of PBS.

### Histopathology

Histological samples were collected at the time of necropsy. 5 µm sections were cut from formalin fixed paraffin embedded tissues and stained with hematoxylin and eosin. A trained pathologist (MNX) performed histopathology scoring in a blinded fashion to quantify inflammatory lesions in liver tissue.

### *In vivo* antibody-mediated anemia and administration of recombinant IL-10

Uninfected mice were administered IP either 600 µg of rabbit anti-mouse RBC IgG (Rockland) in 0.2 ml PBS at Days 4, 6, 10 and 11 or 3 µg of mouse IL-10 recombinant protein (eBioscience) in 0.1 ml PBS every 12 hours starting at Day 8.5 to Day 13.5, or both treatments combined. Control mice received 600 µg of non-specific rabbit IgG (Rockland) in 0.2 ml PBS, or 0.1 ml PBS concurrently.

### Liver CD11b^+^ cell isolation

Cells were isolated using CD11b MicroBeads (Miltenyi Biotec) according to the manufacturer's protocol. Briefly, gallbladders were removed aseptically, and livers were digested with collagenase type II to obtain a single cell suspension. Hepatocytes were removed by centrifugation and CD11b^+^ cells were positively selected by a magnetic column.

### Flow cytometry

Flow cytometry analysis was performed for the detection of neutrophils in livers from *S.* Typhimurium and co-infected CBA mice 4 days post IG bacterial infection. Single cell suspensions of liver tissue were obtained according to protocol described by Miltenyi Biotec for the preparation of single-cell suspensions from mouse liver. Briefly, livers were perfused with 10 mL of PBS (GIBCO) by cardiac puncture, gallbladders were removed aseptically, and livers were digested with collagenase type IV for 30 minutes at 37°C. Tissue was dissociated using a gentleMACS Dissociator (Miltenyi Biotec) and passed through a 70 µm filter. Hepatocytes were removed by centrifugation and red blood cells were lysed using ACK buffer (Lonza). Liver cells were resuspended in PBS and counted. 4×10^6^ cells/mouse were resuspended in 2 mL of PBS and stained with Aqua Live/Dead cell discriminator (Invitrogen) according to the manufacturer's protocol. After Live/Dead staining, cells were washed with PBS and resuspended in 50 µL of PBS containing 1% bovine serum albumin and 2 mM EDTA (fluorescence-activated cell sorter [FACS] buffer). Cells were stained with a FC receptor blocking antibody, anti-CD16/CD32 (eBioscience), for 5 minutes at 4°C and then stained with a cocktail of anti-B220 PE (BD Biosciences), anti-CD3 PE (BD Biosciences), anti-NK1.1 PE (Biolegend), anti-CD11b PECy7 (eBioscience), and Ly6G PerCp/Cy5.5 (Biolegend) for 30 minutes at 4°C. Cells were washed in FACS buffer, fixed with 4% formaldehyde for 30 minutes at 4°C, and then resuspended in FACS buffer. 50 µL of SPHERO AccuCount Fluorescent Particles 10.1 µm (Spherotech) were added to each sample prior to analysis in order to determine absolute counts. Calculation of absolute counts was performed according to manufacturer's protocol. Flow cytometry analysis was performed using an LSRII apparatus (Becton Dickinson), and 7.5×10^5^ events were collected per mouse. Resulting data were analyzed using FlowJo software (Treestar, inc. Ashland, OR). Gates were based on Fluorescence-Minus-One (FMO) controls.

### Statistical analysis

The statistical significance of differences between groups was determined by a Student's *t* test or one-way ANOVA on logarithmically transformed data. For data sets in which all values were the same (zero), statistical significance was determined by a Mann-Whitney U test. A *P* value of 0.05 or less was considered to be significant. All data were analyzed using two-tailed tests.

## Supporting Information

Text S1 Supplementary methods, table of primers used for qRT-PCR and Figures S1–S6.
(PDF)
