# Correction: Malaria Parasite Infection Compromises Control of Concurrent Systemic Non-typhoidal *Salmonella* Infection via IL-10-Mediated Alteration of Myeloid Cell Function

**DOI:** 10.1371/journal.ppat.1004330

**Published:** 2014-07-25

**Authors:** The PLOS Pathogens Staff

There is a missing affiliation for the third author, Brian P. Butler. His correct affiliations are: Department of Microbiology & Immunology, School of Medicine, University of California at Davis, Davis, California, United States of America and School of Veterinary Medicine, St. George's University, Grenada, West Indies
