# Supplementary material for: Malaria Parasite Infection Compromises Control of Concurrent Systemic Non-typhoidal Salmonella Infection via IL-10-Mediated Alteration of Myeloid Cell Function
Source: PLoS Pathog. 2014 May 1;10(5):e1004049. doi: 10.1371/journal.ppat.1004049 (PMC4006898; doi:10.1371/journal.ppat.1004049)
Supplement: Text S1 — Supplementary methods, table of primers used for qRT-PCR and Figures S1–S6. (PDF) [file ppat.1004049.s001.pdf]

Supporting Information S1 for Lokken, Mooney et al, *Malaria parasite infection compromises control of concurrent systemic non-typhoidal Salmonella infection via IL-10-mediated alteration of myeloid cell function*, published 2014 in PLoS Pathogens

---

- Supplementary Methods
- Table: qRT-PCR primers used in this study
- Supplementary Figures 1-6

Supplementary Methods:

***In vivo* IL-10 depletion**. Co-infected mice were administered i.p. 300 µg, 200 µg and 100 µg of rat anti-mouse IL-10 IgG1 kappa (eBioscience) in 0.1 ml PBS at Days 9, 10 and 11, respectively. Control mice received 300 µg, 200 µg and 100 µg of rat IgG1 kappa (BD Pharmingen) in 0.1 ml PBS concurrently.

Microbial readouts of infection. Parasitemia was determined by counting the percentage of *Plasmodium yoelii* iRBCs on thin blood smears stained with Giemsa (Acros Organics). Plasma IL-10 levels were determined by ELISA (eBiosciences) following the manufacturer's protocol. To determine the numbers of viable *Salmonella*, cecal contents, Peyer's patches (3 per mouse), mesenteric lymph nodes, livers, and spleens were homogenized in PBS using an Ultra Turrax T25 Basic mixer (IKA). Blood was collected from the heart with heparinized needles, plasma removed, then incubated for 10min with 120µL 1% triton x-100 in PBS. Homogenates were serially diluted and plated on LB agar plates containing 100mg/L nalidixic acid (Sigma). After overnight growth at 37°C, CFU/gram of tissue was calculated.

RNA extraction, reverse transcription-PCR (RT-PCR), and real-time PCR.
Animal tissues were frozen in liquid nitrogen at the time of necropsy and stored at -80°C. RNA was extracted from tissue as described previously (Roux, 2007) using Tri-Reagent (Molecular Research Center) according to the instructions of the manufacturer. All RNA was treated with DNAseI (Ambion) to remove genomic DNA contamination. For a quantitative analysis of mRNA levels, 1µg of total RNA from each sample was reverse transcribed in a 50-µl volume (TaqMan reverse transcription [RT] reagent; Applied Biosystems), and 4 µl of cDNA was used for each real-time reaction. RT-PCR was performed using the primers listed in Table 1, SYBR green (Applied Biosystems) and ViiA 7 Real-Time PCR System (Applied Biosystems). Data was analyzed by using the comparative threshold cycle (C_T) method (Applied Biosystems). Target gene transcription of each sample was normalized to the respective levels of GAPDH mRNA and represented as fold change over gene expression in control animals or absolute quantification was determined using gene-specific plasmid standards in each run.

Isolation of neutrophils from mouse bone marrow. Bone marrow fractionation was performed using by a modification of the Percoll gradient centrifugation method described by Boxio et al. (1,2). Bone marrow was flushed from femora and tibiae with HBSS prep (10X HBSS Ca-Mg free diluted to 1X with 20mM Na-HEPES (pH 7.4) and 0.5% FCS) and passed through an 18-gauge needle twice to disrupt larger bone marrow clumps and cells were pelleted by centrifugation at 400 x g. Red blood cells were lysed by resuspension of cells in 0.2% NaCl, then the concentration adjusted to 1.2% NaCl. Cells were passed through a 70 micron strainer and pelleted at 400 x g. The resulting pellet was re-suspended in 5ml HBSS prep. The cell suspension was layered onto a three-layer percoll (Sigma) gradient (78% /69% /52%) and centrifuged at 1000 x g for cell separation. The band at the interface of 69%/78% percoll layers was carefully collected, washed twice with HBSS prep and used for further experiments. The composition of the cell population was confirmed by microscopy to have neutrophil morphology and by and flow cytometry to contain predominantly CD11b$^+$ Ly6G$^+$ cells.

Table: Quantitative real-time PCR (qRT-PCR) primers used in this study.

**Target gene**	**Method**	**Sequences (5'-3')**
Gapdh	Fold-change	TGTAGACCATGTAGTTGAGGTCA<br>AGGTCGGTGTGAACGGATTTG
Il10	Fold-change &<br>Absolute copies	GGTTGCCAAGCCTTATCGGA<br>ACCTGCTCCACTGCCTTGCT
Cxcl1 (Kc)	Fold-change &<br>Absolute Copies	GCTTGCCTTGACCCTGAAGCTC<br>TGTTGTCAGAAGCCAGCGTTCAC
Lcn2	Fold-change &<br>Absolute Copies	ACATTTGTTCCAAGCTCCAGGGC<br>CATGGCGAACTGGTTGTAGTCCG
Gapdh	Absolute Copies	TCATCAACGGGAAGCCCA<br>AGACTCCACGACATACTCA

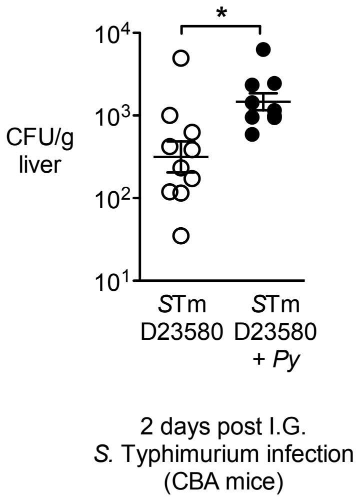

Figure S1, related to Figure 1:

Concurrent malaria increases systemic colonization with a Malawian clinical NTS isolate. CBA mice were inoculated IP with blood stage *P. yoelii* as in Fig 1A, and at 10 days mice were inoculated IG with *S.* Typhimurium clinical isolate D23580. 2 days after *S.* Typhimurium infection bacterial colonization of the liver was determined. Dots represent individual mice and bars represent the mean CFU ± SEM (n=10). Statistical significance was determined using an unpaired Student's *t* test on log-transformed values and is indicated as *, $P < 0.05$; **, $P < 0.01$.

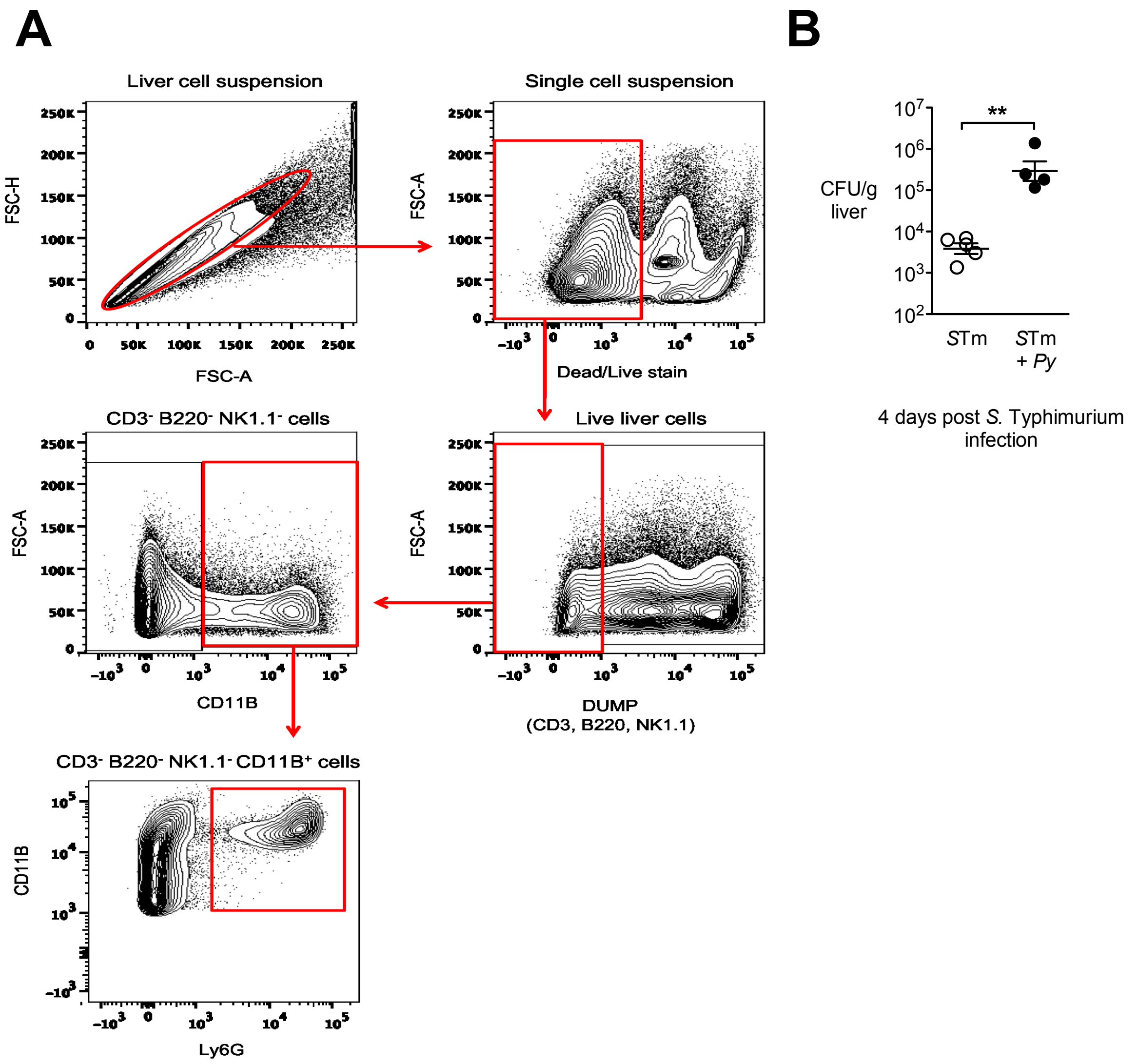

Figure S2, related to Figure 2:

A, Gating strategy for analysis of liver single cell suspensions. After doublet elimination, live cells were gated and $CD3^-$ $B220^-$ $NK1.1^-$ cells were analyzed for CD11b and Ly6G expression. Neutrophils were defined as $CD11b^+$ $Ly6G^+$. **B,** CFU of *S.* Typhimurium in the liver 4 days after IG infection of CBA mice (n=4-5). Results are from experiment shown in figure 2D. Dots represent individual mice and bars represent ± SEM. Statistical significance was determined using an unpaired Student's *t* test on log-transformed values and is indicated as *, $P < 0.05$; **, $P < 0.01$

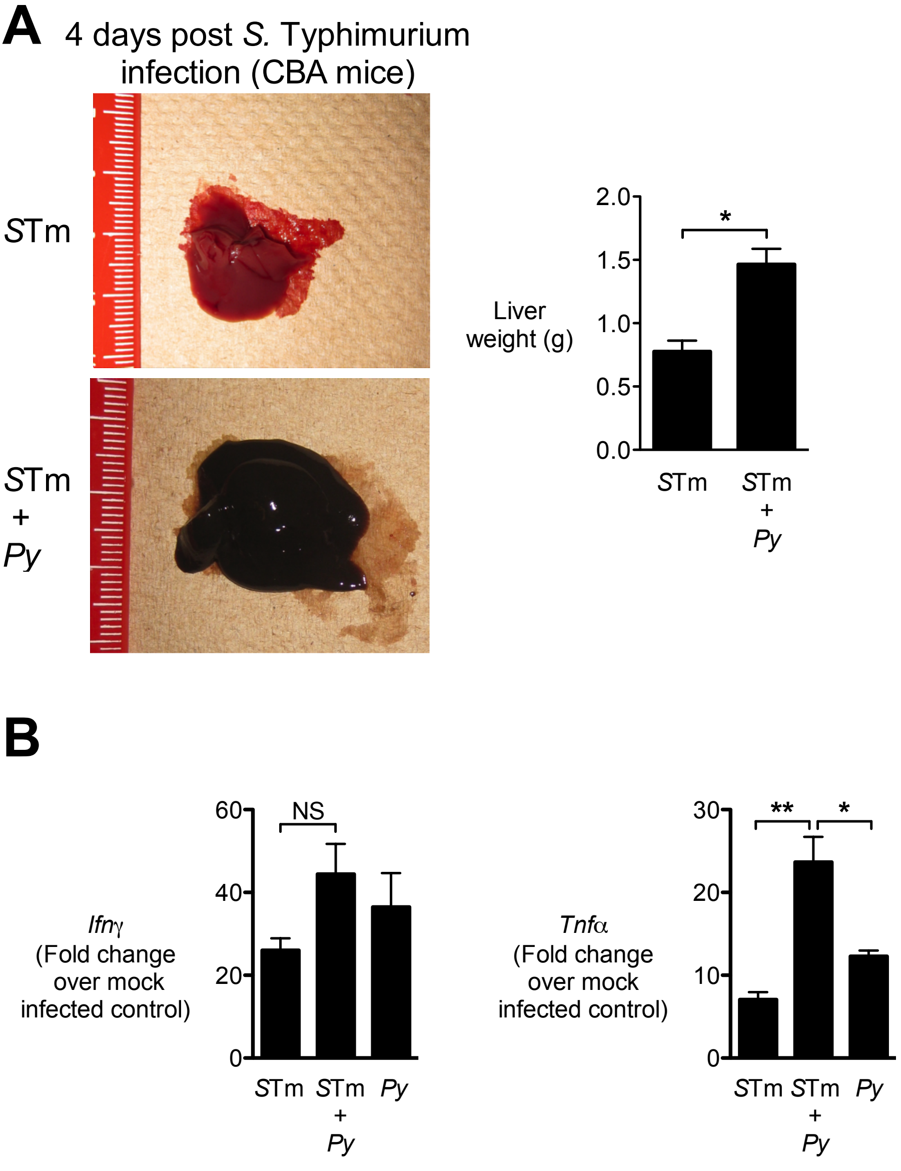

Figure S3, related to Figure 2:

A, Representative photographs of livers from *S.* Typhimurium and co-infected CBA mice 4 days post-*S.* Typhimurium infection. Right panel represents the mean liver weight (grams) from *S.* Typhimurium and co-infected CBA mice (n=4-5). **B,** Expression of *Ifnγ and Tnfa* from the livers of *P. yoelii* (*Py*), *S.* Typhimurium and co-infected CBA mice, 4 days post-*S.* Typhimurium infection (n=4-9). Data are expressed as fold change over mock-infected control. Results are from experiment shown in figure 1B. Statistical significance was determined using an unpaired Student's *t* test on log-transformed values and is indicated as *, $P < 0.05$; **, $P < 0.01$.

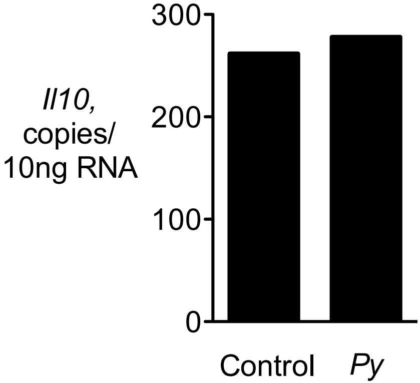

Figure S4, related to Figure 7:
Expression of *Il10* in neutrophils enriched from the bone marrow of C57BL/6 mice that were uninfected (control; n=3) or infected with *P. yoelii* (Py; n=4).

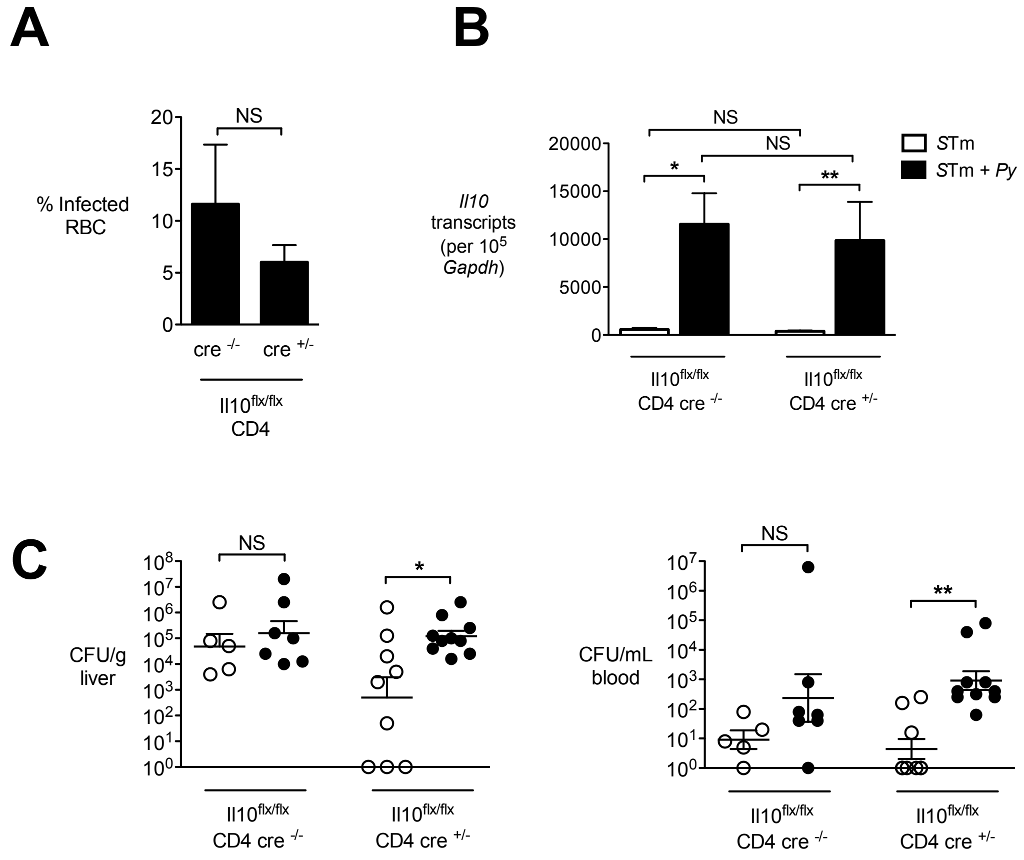

Figure S5, related to Figure 7:

Co-infection with *S.* Typhimurium in mice conditionally deficient for IL-10 production by CD4 T cells (*$Il10^{f/f}$ CD4-cre)* (n=5-9). **A,** Parasitemia in conditionally IL-10 deficient mice and Cre-negative controls **B,** Expression of *Il10* in livers of *$Il10^{f/f}$ CD4-cre* mice or littermate controls at 12 after *P. yoelii* infection and 2 days after *S.* Typhimurium infection. Bars represent the mean +SEM (n=5-10). **C,** *S.* Typhimurium infection and co-infection of mice conditionally deficient for IL-10 production by T cells (*$Il10^{f/f}$ CD4-cre)* or littermate controls. Mice were co-infected as described in Fig. 1A and bacterial colonization was assessed 2 days after IP inoculation with *S.* Typhimurium (n=5-10). Data are compiled from 2 independent experiments. Dots represent individual mice and bars represent ± SEM. Statistical significance was determined using an unpaired Student's *t* test on log-transformed values and is indicated as *, $P < 0.05$; **, $P < 0.01$.

Liver During Malaria & *Salmonella* Coinfection

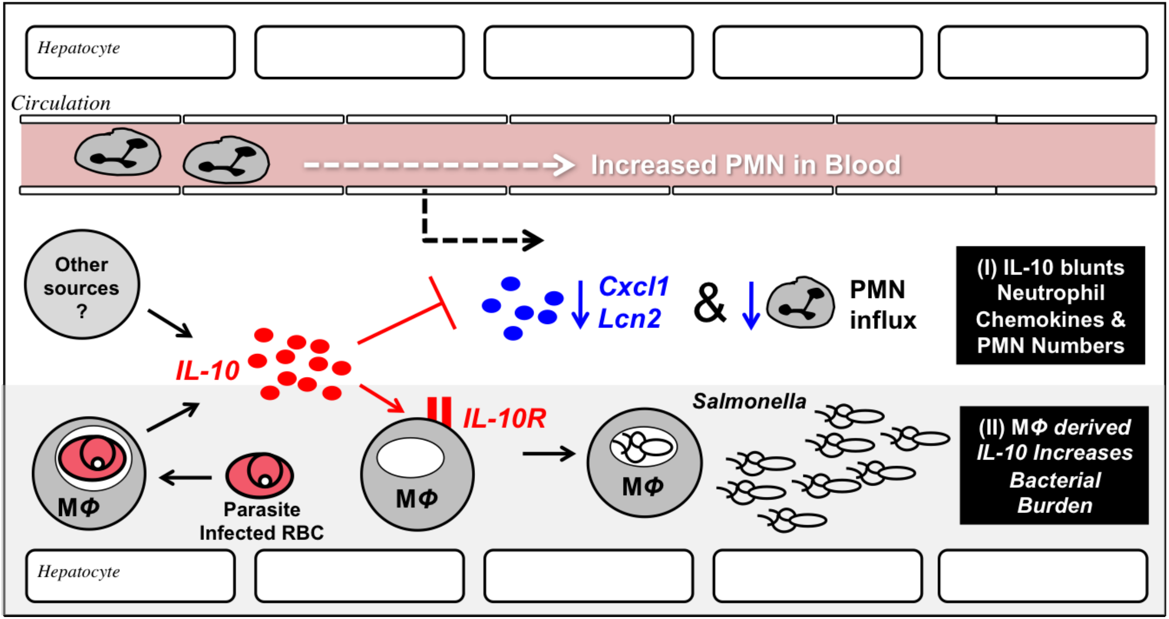

Figure S6: Proposed model describing the effect of malaria parasite induced IL-10 on the immune response to *S.* Typhimurium during co-infection in the liver. Malaria parasite infection increases the production of IL-10 by myeloid cells (most likely macrophages) in the liver. Increased local levels of IL-10 blunt expression of neutrophil chemokines, *Cxcl1* and *Lcn2*, and reduce neutrophil infiltration. IL-10 also acts on phagocytic cells and alters their phenotype to render them more permissive to *S.* Typhimurium infection.
